# Quality of life and its associated factors among women with pelvic organ prolapse who attend gynecology clinics Southern Ethiopia 2022

**DOI:** 10.1186/s12905-024-03238-1

**Published:** 2024-07-12

**Authors:** Ayenew Tega, Fentahun Yenealem, Getahun Belay, Eden Asmare, Temesgen Getaneh, Misgana Desalegn, Natnael Dechasa, Zemenu Addis

**Affiliations:** 1 Department of Midwifery, Hosanna health science colleg, Hosanna, Ethiopia; 2 Department of Midwifery, College of Medicine and Health Science, Bahir Dar University, Bahir Dar, Ethiopia; 3 Department of Midwifery, College of Medicine and Health Science, Debre Markos University, Debre Markos, Ethiopia; 4 Department of Midwifery, Hossana College of Health Science, Hossana, Ethiopia; 5 Department of Midwifery, College of Medicine and Health Science, Dire Dawa University, Dire Dawa, Ethiopia; 6 Department of Nursing, Hosanna health science colleg, Hosanna, Ethiopia

**Keywords:** Quality of life, Pelvic organ prolapse, Ethiopia

## Abstract

**Introduction:**

Pelvic organ prolapse is the implosion of one or more pelvic floor structures which affect womens quality of life by compromising overall health, physical, social, structural, functional and emotional well-being.

**Objective:**

To assess the quality of life and its associated factors among women with pelvic organ prolapse who attend gynecology clinics at Gurage zone hospitals, Southern Ethiopia 2022.

**Methods:**

Facility-based cross-sectional study was applied in gurage zone hospital from April, 30 to Jun 30, 2022. Systematic random sampling was employed to select 416 women. Interview based structured questionnaires were applied to collect the data. The collected data were analyzed using Statistical Produte and Service Solution. Binary and multivariable logistic regressions were fitted to assess the association between dependent and independent variables. *P*-value < 0.05 was used to declare the final statistical significance.

**Result:**

The mean (SD) score of quality of life in this study was 53.57 (21.59). The most affected domains were general health perception and physical limitation (mean (SD) score 67.45 29.24) and (64.26 32.36)) respectively. Had no formal education (AOR = 1.50, 95% CI: 1.02, 3.12), stage III/IV POP (AOR = 2.02, 95% CI: 1.19, 3.60), constipation (AOR = 3.51, 95% CI: 2.12, 7.21), urge urinary incontinence (AOR = 3.89, 95% CI: 2.32, 6.95), and not did regular physical exercise (AOR = 2.18, 95% CI: 1.41, 3.37) were significantly associated with poor quality of life.

**Conclusion:**

More than half of the participants in this study had impaired quality of life. The factor associated with quality of life was had no formal education, stage III/IV, constipation, urge urinary incontinence, and regular physical activity. It is recommended to have access education, counseling regular physical activity, detection, and management of its comorbidity.

**Supplementary Information:**

The online version contains supplementary material available at 10.1186/s12905-024-03238-1.

## Background

Pelvic organ prolapse (POP) is a multifaceted condition resulting from the dimness and defects of pelvic floor structures [1]. Pelvic organ prolapse is the descent of one or more pelvic organs via the vaginal canal, including the vagina, uterus, rectum, bladder, cervix, post-hysterectomy vaginal cuff, and small or large bowel. [2, 3]. Worldwide prevalence of pelvic organ prolapse has recently been reported to be around 9% [2]. POP is caused by both internal and extrinsic factors, including parity, a history of previous hysterectomy, co-morbidities, occupation, age, postmenopausal status, and intra-abdominal pressure [4–6]. POP increase with age and high parity was the single most important risk factor for POP among women in rich as well as poor countries [7, 8]. In postmenopausal women, it causes considerable quality of life degradation due to a variety of symptoms and recurring surgical procedures, and it has a negligible mortality and morbidity rate. It affects about half of all women over the age of 50 [9].

In Sub-Saharan Africa, the effects of POP on women’s quality of life were more severe [7]. Patients report difficulty sitting, moving, and lifting, along with a lump in their vagina. In the Gurage zone, Ethiopia, there were high burdens of pelvic organ prolapse, with overall 41.1% of the participants reporting one or more symptoms of pelvic floor disorders, followed by a prevalence of pelvic organ prolapse (25.5%). The most common cause was old age, long hours of carrying heavy objects, a high parity, a history of home delivery, and a history of chronic constipation [10]. Similarly in Ethiopia, it affects 23.52% of women [3].

Women with POP live with a variety of symptoms including sleep, physical, urinary, bowel, sexual, and psychological or emotional disterbance [11, 12]. Advanced stage POP symptoms negatively impact women’s quality of life by social rejection, self-esteem loss, depression, restlessness, bowel irritability, energy loss, sleep disturbance, and reproductive and urinary tract infections [13–15]. Rural women with POP often divorce due to social shame and fear of sharing their difficulties, particularly in daily tasks like childcare, cooking, fetching water, and farming [16, 17].

Dependent on factors including economic status, way of life, educational attainment, and cultural background, women with POP have varying quality of life in different countries [18]. Furthermore, by imposing limitations on physical, social, and sexual activities, causing psychological discomfort, and raising the financial burden associated with health care, it has the potential to significantly impair women’s quality of life about their health [16, 17]. However, with the expense of POP operations and the lack of qualified doctors, access to health care is extremely limited in low-income nations [19].

Compared to developed nations, women’s quality of life is significantly impacted by pelvic organ prolapse in developing nations [20]. Because there is insufficient data on the quality of life associated with pelvic organ prolapse, Ethiopia and other developing countries lack access to the pelvic organ prolapse-related QoL tool, which is used in developed countries to assess the quality of life and its associated factors in women with POP and establish a baseline strategy for treatment [21, 22]. One of the causes of extreme morbidity and psychological distress for the patient, who is frequently socially reclusive and stigmatized is pelvic organ prolapse, which also has a detrimental impact on the socioeconomic status and reproductive behavior of affected women [23, 24].

However, in Ethiopia, the preoperative quality of life study among women who had POP is not well understood [25], and no research was done in the study area specifically. Therefore, measuring the quality of life and its associated factors among women who had pelvic organ prolapse has theoretical as well as practical significance for a health care provider, program planner, and policymakers to use as baseline data to focus on the basic factors and to develop a feasible intervention plan that makes preoperative care.

## Materials and methods

### Study area, population and period

A facility-based cross-sectional study was conducted in Gurage Zone Hospital, Southern Ethiopia. It is bordered on the southeast by Hadiya, on the west Yem special woreda, on northeast Oromia Region, and by the southeast Silt’s Zone. According to the 2018/2019 annual report of the Gurage zone health office, there are seven hospitals and 72 health centers serving the total population of the zone. Among these hospitals, five are primary hospitals, one general hospital, and one compressive specialized hospital. All hospitals deliver comprehensive obstetrical and gynecological care [26]. The study was employed from April 30 to Jun 30 in all hospital found at Gurage Zone Hospital, Southern Ethiopia 2022. All women who had pelvic organ prolapse in gynecologic clinics at Gurage Zone Hospitals were included in this study. Being pregnant and having a pelvic cancer diagnosis (including ovarian, endometrial, cervical, and endometrial fibroid) were excluded from the study.

### Sample size and sampling procedure

The sample size was calculated by using the single population proportion formula.

$${{\text{(Za/2)}}^{\text{2}}}{\text{p(1 - p)/}}{{\text{d}}^{\text{2}}}$$

**Fig. 1:**
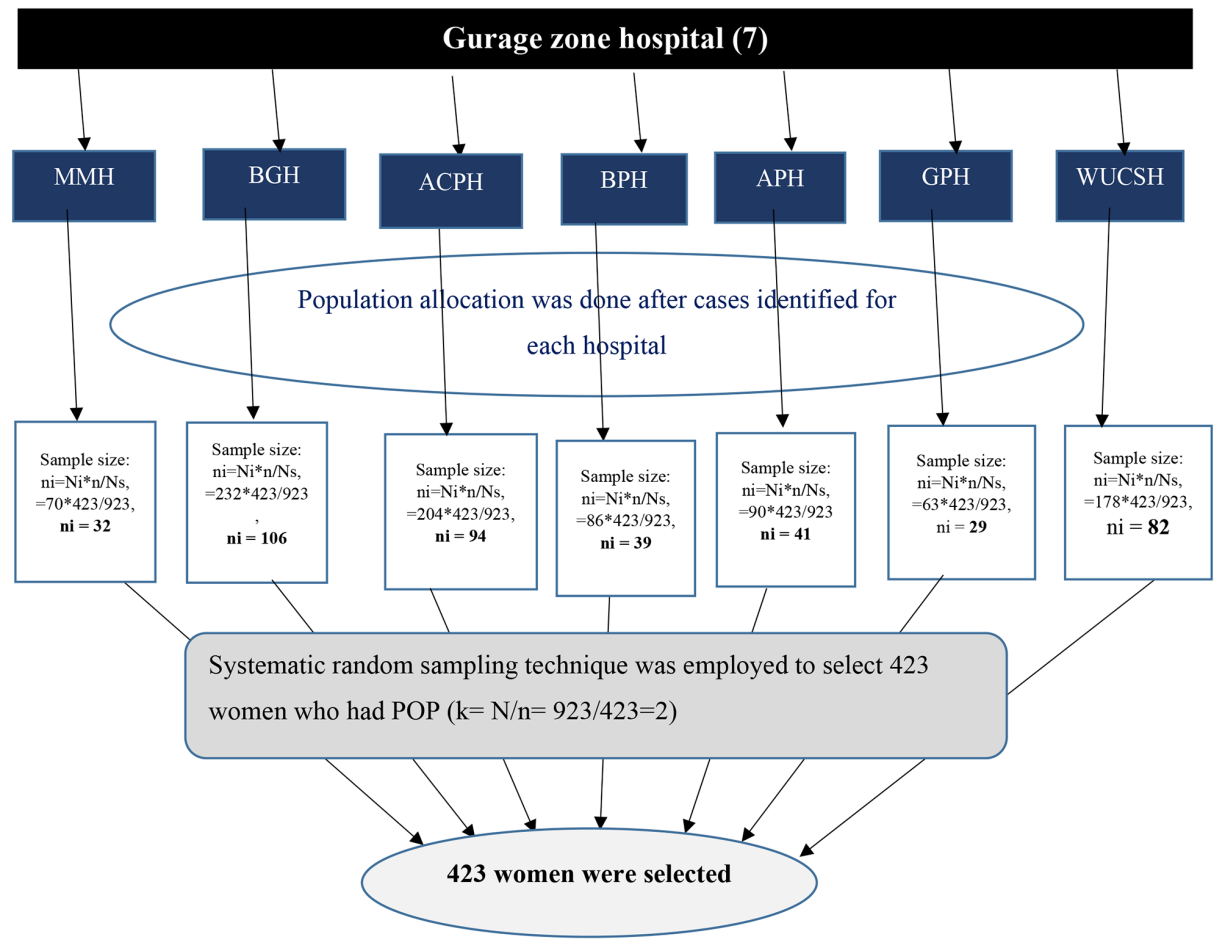
Schematic presentation of sampling procedure to select 423 women, at Gurage Zone hospitals, Southern Ethiopia 2022. Note: MMH: Mehal Meda Hospital, BGH: Butajira General Hospital, ACPH: Atate Catholic Primary Hospital, BPH: Buie Primary Hospital, APH: Agena Primary Hospital, WUSCH: Wolkite University specialized and compressive Hospital, GPH: Gunchire Primary Hospital

Where = required sample size for a very large population (*N* > 10,000), Z = critical value for.

normal distribution the 95% confidence level which equals 1.96 at α = 0.05, P = prevalence of poor QoL, and d = 0.05 (5% margin of error). With the assumptions of 95% CI, 5% established the prevalence of poor quality of life among POP women at 50% (*P* = 0.5) the formula yields = 384. The stated p-value was selected to get the maximum sample size and give 423 with a 10% non-response rate. All hospitals found in Gurage Zone (5 public hospitals and 2 non-governmental hospitals) were selected for this study. The total sample size was proportionally allocated for each hospital based on the previous two months’ pelvic organ prolapse report (February 1, 2022, to March 30, 2022). A systematic random sampling technique was used to select study participants. The sampling interval (K) was calculated by K = N/n, where N = the total average number of mothers with POP expected to attend a gynecologic clinic in each hospital, n = proportionally allocated sample size in each hospital, which yields 923/423 = 2 (Fig. 1). Then, a similar k-value (2) was used to select each patient. The first study participant was selected using a simple random sampling technique via the lottery method among the first two mothers who had confirmed pelvic organ prolapse in the gynecologic clinic on the first day of data collection. Then, every second mother was enrolled in the study based on the sequence of their diagnosis of POP, and before the operation.

### Definition of outcome

**Poor quality of life in each domain** indicates participants who scored greater than or equal to median value in each domain [11, 17].

**Good quality of life in each domain** indicates participants who scored below the median [11, 17, 27].

**Overall good quality of life is** measured by a score below the median of the overall (nine) QoL domains [11, 17, 27, 28].

**Overall poor quality of life** measured by a greater or equal to the median score of the overall (nine) QoL domains [11, 17, 27, 28].

**Regular physical exercise** is considered a regular movement within a week which might be a physician-ordered or self-provided activity (includes **aerobic exercise** such as walking for a minimum of 30 min, swimming, seated cycling, recreational activity, and **pelvic floor exercise** (kegals’ exercise)) [29–31].

### Data collection tool and procedure

A data collection tool was developed after reviewing different related articles [17, 27, 32]. P-QoL among women with POP was evaluated using the quality of life measurement tool, which is validated in Ethiopia, after translation into the Amharic language [33], and into the Gurage language. All questions, except the first, which has five points, are assessed on a four-point scoring system (0 = not at all, 1 = slightly, 2 = moderately, 3 = a lot). The items were attributed to 9 domains that were transformed into a scale of 0 = (good HRQoL) excellent to 100 = ( HRQoL) poor: general health perception (one item: 1), prolapse impact (one item: 2), role limitation (two items: 3–4), physical limitation (two items: 5–6), social limitation (two items: 7–8), personal relationships (three items: 9–11), emotions (three items: 12–14), sleep/energy (two items: 15–16), and severity measurement (four items:17–20). The overall QoL was obtained as an average score of the total nine domains. The higher the score the poorer the QoL [27, 33]. First, the tool was prepared in an English version. Then, it was translated to the Amharic version, and finally to the Gurage local language. Finally, it was translated back to the English version to check its consistency. Interview and chart review (POP stage) based structured questionnaires were applied to collect the data. The data collector gets the confirmed diagnosis at the gynecological outpatient department, and data collection was done at the time of confirmed diagnosis. Mother’s Sociodemographic data, general health conditions, obstetric and gynecological, and pelvic floor symptom-related data were included in the tool. Fourteen diploma and above midwives and Six senior supervisors who had experience in similar work were recruited as data collectors and supervisors respectively.

### Data quality assurance

The tool was pretested from 22 women in Kibet Primary Hospital to ensure consistency and completeness of the questionnaire. The one-day training was given to both data collectors and supervisors by a principal investigator about the objective of the study, data collection tool, procedure, and how to fill out the questionnaires. Data collectors were supervised throughout the data collection period. Then, the overall process was coordinated and controlled by the principal investigator.

### Data analysis

The collected data were entered using the Epi data version 3.1 computer program. Then, it was exported to Statistical Package of Social Sciences version 25 for analysis. Descriptive statistics like frequency and summary statistics were employed to describe the characteristics of the study participants. The outcome variable, i.e. QoL, was dichotomized into good and poor based on the analyzed mean scores. A logistic regression model was also fitted to determine if there is any association between QoL and the independent variable. All explanatory variables in binary logistic regression with a *p*-value of 0.2 or less were considered for multivariable logistic regression analysis. The adjusted odds ratio with their corresponding 95% confidence intervals and p-value less than 0.05 was used to declare the association between dependent and independent variables and statistical significance in this study. Then, multivariable logistic regression using the backward likelihood ratio method was applied to explore variables in the final model or step. Furthermore, model fitness was tested using the Hosmer and Lemeshow Goodness of Fit test with a p-value of 0.549. In addition, it was confirmed that there was no problem of interaction effect and multicollinearity among independent variables, with a variance inflation factor of < 2 for all variables.

## Result

### Socio-demographic characteristics

A total of 416 mothers with POP participated in this study, yielding a response rate of 98.5%. The mean (± SD) age of the respondent was 46.94 (± 10.04) years, ranging from 26 to 68 years. The majority of the respondents, 345 (82.9%) resided in rural areas. Regarding the educational status of participants, 269 (64.7%) were not formally educated, and 73 (17.5%) had primary education. Concerning the occupation of the participants, 263 (63.2%) were farmers and 84 (20.2%) were housewives. (Table 1).

**Table 1:** Socio-demographic characteristics of women with pelvic organ prolapse in Gurage Zone Hospitals, Southern Ethiopia: 2022 (*n* = 416)

Variables	Category	Frequency	Percent (%)
Age	26–34	52	12.5
	35–49	183	44.0
	> 49	181	43.5
Residence	Urban	71	17.1
	Rural	345	82.9
Marital status	Married	317	76.2
	Single	7	1.7
	Divorced	45	10.8
	Widowed	47	11.3
Maternal education	Had no formal education	269	64.7
	Primary education	73	17.5
	Secondary and above	74	17.8
Ethnicity	Gurage	393	94.5
	Amhara	14	3.4
	Siltie	9	2.1
Religion	Muslim	204	49
	Orthodox	171	41.1
	Protestant	26	6.3
	Catholic	15	3.6
Maternal occupation	Housewife	84	20.2
	Farmer	263	63.2
	Government employ	22	5.3
	Private employ	17	4.1
	Merchant	30	7.2
Husband occupation	Farmer	185	58.4
	Government employ	40	12.6
	Private employ	21	6.6
	Merchant	71	22.4
	*≤* 2000	114	27.7
Family monthly income(ETB)	2001–3999	122	29.3
	*≥* 4000	180	43

### General health characteristics

Out of 416 respondents, only 121 (29.1%) women did physical exercise at least once per week. Twenty-one (5%) of the respondents were overweight, and thirty-one (7.5%) of the respondents had hypertension (Table 2).

**Table 2:** General health characteristics of women with pelvic organ prolapse in Gurage Zone Hospitals, Southern Ethiopia: 2022 (*n* = 416)

Variables		Frequency	Percent (%)
BMI	Underweight	29	7.0
	Normal weight	366	88.0
	Overweight	21	5.0
Regular physical exercise (at least 1/week)	Yes	121	29.1
	no	295	70.9
Self-reported depression	Yes	117	28.1
	No	299	71.9
Hypertension	Yes	31	7.5
	No	385	92.5

### Obstetrics and gynecologic characteristics

Concerning obstetrics and gynecology characteristics, more than half of the study participants, 232 (55.8%), were in menopause. In addition, out of 416 respondents, 98 (23.6%) had a previous history of hysterectomy, and 269 (65.8%) were grand multigravida. (Table 3).

**Table 3:** Obstetrics and gynecologic characteristics of women with pelvic organ prolapse in Gurage Zone Hospitals, Southern Ethiopia: 2022 (*n* = 416)

Variables		Frequency	Percent (%)
Gravidity	Primigravida	9	2.2
	Multigravida	131	32
	Grand multigravida	269	65.8
Parity	Primipara	9	2.2
	Multipara	167	40.8
	Grand multipara	233	57.0
Menopause	Yes	232	55.8
	No	184	44.2
Previous hysterectomy	Yes	98	23.6
	No	318	76.4
Anal surgery	Yes	27	4.5
	No	389	95.5
Urinary incontinence surgery	Yes	81	19.5
	No	335	80.5

### Pelvic floor symptom characteristic

Out of 416 respondents, 238 (57.2%) women had advanced stages (III/IV) of POP. One hundred eighteen (28.4%) of the respondents had urge urinary incontinence, 122 (29.3%) had constipation, 110 (26.4%) felt a bulge or lump, and 131 (31.5%) had pain or discomfort during sexual intercourse. (Table 4).

**Table 4:** Pelvic organ prolapse stage and pelvic floor symptom of women with pelvic organ prolapse in Gurage Zone Hospitals, Southern Ethiopia: 2022 (*n* = 416)

Variables		Frequency	Percent (%)
POP Stage	Stage I-II	178	42.8
	Stage III-IV	238	57.2
Urge urinary incontinence	Yes	118	28.4
	No	298	71.6
Stress urinary incontinence	Yes	121	29.1
	No	295	70.9
Frequent urination	Yes	107	25.7
	No	309	74.3
Difficulty in emptying bladder	Yes	151	36.3
	No	265	63.7
Urinary urgency	Yes	101	24.3
	No	315	75.7
Lower abdominal pain or heaviness	Yes	126	30.3
	No	290	69.7
The feeling of a bulge in vagina	Yes	110	26.4
	No	306	73.6
Pain or discomfort during sexual intercourse	Yes	131	31.5
	No	285	68.5
Bowel movement	Yes	33	7.9
	No	383	92.1
Constipation	Yes	122	29.3
	No	294	70.7
Fecal incontinence	Yes	46	11.1
	No	370	88.9

### Prolapse-related quality of life

In this study, the overall pelvic organ prolapse-related quality of life mean score of participants was 53.57 (95% CI: 51.49, 55.65). In addition, the prolapse-related poor QOL among women who had POP was 51.68% (95% CI: 46.86, 56.47). The most affected domain was general health perception, with a mean (± SD) score of 67.45 (± 29.24) while the list affected was severity measure with a mean (± SD) score of 25.66 (± 23.34). (Table 5).

**Table 5:** Pelvic organ prolapse-related quality of life domain score of women with pelvic organ prolapse in Gurage Zone Hospitals, Southern Ethiopia: 2022 (*n* = 416)

Quality of life domain	Mean	± SD
General health perception	67.45	29.24
Prolapse impact	61.85	33.74
Role limitation	62.62	34.86
Physical limitation	64.26	32.36
Social limitation	62.66	33.57
Personal relation	56.27	31.47
Emotion	53.39	28.34
Sleep/ energy	27.96	30.00
Severity of measurement	25.66	23.34
**Overall average score**	**53.57**	**21.59**

### Factors associated with poor quality of life among women with POP

In bivariate analysis, maternal educational status, regular physical exercise, menopause, urge to urinary incontinence, POP stage, previous hysterectomy, feeling of bulging, discomfort during sexual intercourse, and constipation were significantly associated with poor quality of life at a p-value ≤ 0.2.

In logistic regiration analysis, having no formal education, doing regular physical exercise at least once per week, POP stage III/IV, urge urinary incontinence, and constipation remained significantly associated with poor quality of life at a p-value of less than 0.05. (Table 6).

According to the present regression, the likelihood of having poor quality of life among women who had no formal education with pelvic organ prolapse was 1.50 times more likely when compared with women who had secondary education and were above the educational level (AOR = 1.50, 95% CI: 1.02, 3.12). The likelihood of having poor quality of life among women who didn’t do regular physical exercise was 2.18 (AOR: 2.18, 95% CI: 1.41, 3.37) times more likely than their counterparts. In addition, the likelihood of having poor quality of life among women who had POP stages three and four was 2.02 (AOR = 2.02, 95% CI: 1.19, 3.60) times higher than for women who had POP stages one and two. Furthermore, women who had experienced urge urinary incontinence were 3.89 times more likely to have a poor quality of life compared with their counterparts (AOR = 3.89, 95% CI: 2.32, 6.95). Moreover, the odds of poor quality of life among women who had constipation were 3.51 times higher when compared with their counterparts (AOR = 3.51, 95% CI: 2.12, 7.21). (Table 6).

**Table 6:** Factors associated with quality of life among women with pelvic organ prolapse in Gurage Zone Hospitals, Southern Ethiopia: 2022 (*n* = 416)

Variables		Quality of life				
		Frequency (%)		COR (95% CI)	AOR (95% CI)	p-value
		Poor	Good			
Maternal educational status						
	Had no formal education	157(57.9)	112(42.1)	1.56(0.93, 2.61)	1.50(1.02, 3.12)	0.041*
	Primary education	23(31.5)	50(68.5)	0.51(0.26, 1.02)	0.49(0.29, 1.25)	**0.303**
	Secondary and above	35( 49.4)	39(50.6)	1	1	
Regular Physical exercise						
	Yes	46(38)	75(62)	1	1	0.018**
	No	169(57.3)	126(42.7)	2.18 (1.41, 3.37)	1.85(1.11, 308)	
Menopause						
	Yes	128(55.2)	104(41.1)	1.37(0.93 2.02)	1.29(0.92, 2.28)	**0.99**
	No	87(47.3)	97(52.7)	1	1	
Previous hysterectomy						
	Yes	59(60.2)	39(39.8)	1.57(0.99, 2.49)	1.48(0.86, 2.53)	**0.154**
	No	156(49.1)	162(50.9)	1	1	
Urge urinary incontinence						
	Yes	88(74.6)	30(25.4)	3.94(2.45, 6.34)	3.89(2.32, 6.45)	< 0.001***
	No	127(42.6)	171(57.4)	1	1	
POP stage						
	I & II	73(41)	105(59)	1	1	< 0.001***
	III &IV	142(59.7)	96(40.3)	2.12(1.43, 3.15)	2.02(1.19, 3.60)	
Constipation						
	Yes	89(73)	33(27)	3.59(2.26, 5.70)	3.51(2.12, 7.21)	< 0.001***
	No	126(42.9)	168(57.1)	1	1	
Discomfort during sex						
	Yes	76(53.5)	55(42)	1.45(0.95, 2.20)	1.40(0.84, 2.54)	**0.083**
	No	139(48.8)	146(51.2)	1	1	
Feeling of bulging						
	Yes	63(57.3)	47(42.7)	1.35 (0.87, 2.10)	0.93(0.54, 1.60)	**0.811**
	No	152(49.7)	154(50.3)	1	1	

* Significant at *P* < 0.05, ** Significant at *P* < 0.02, *** significant at *p* < 0.001

## Discussion

In this study, the overall pelvic organ prolapse-related quality of life mean score of participants was 53.57 (95% CI: 51.49, 55.65). This finding is higher than the study conducted in Bahir Dar and Uganda [11, 34]. This difference might be due to educational status, target population, assessment tool, and socio-economic characteristics. For instance, a study in Bahir Dar included only symptomatic women, had a small sample size, and only data from one facility in the city. Similarly, a study conducted in Uganda uses King’s health questionnaire which contains seven QoL domains. In relation, this study was in line with a study conducted in Turkey [35], and Slovakia [18]. However, this study was less than that of the studies conducted in the United Kingdom, Italy, and China [36–38]. This variation might be because People’s perceptions of their place in the world are shaped by a variety of factors, including societal ideals, living conditions, cultural norms, educational backgrounds, and personal convictions. Thus, one possible explanation for the variations in QoL scores reported in the various research studies could be the variety of these quality of life assessment reference points among countries [39].

Regarding individual domain scores, general health perception was the most severely affected domain in this study. This result is supported by a study reported in northern Ethiopia [25]. This may be due to women who had POP perceives about their body change, health distorted, isolated, deprived, and feeling less attractive, and it affects their well-being negatively [40].

The physical limitation was the second most affected domain in this study, which was supported by studies done in Bahir Dar [34], Gondar [25], Uganda [11], and Turkey [41]. This might be chronic pelvic pain caused by stretching and weakening of the pelvic ligaments which makes it difficult to walk, bend, and work [32]. This improvement may be the result of the anatomical abnormality being corrected, which would lessen the women’s chronic pain and discomfort and allow them to walk and even work comfortably [42–44]. In this study, the QoL domain score of social life was the third most affected part. This is similar to studies done in Gondar [25], Nepal [45], Uganda [11], and Taiwan [27]. This could be due to the advanced stages of POP being associated with foul-smelling vaginal discharge, and urinary incontinence, leading to social consequences [46]. The social life of the women improved, probably because POP surgery has been found to correct associated urinary incontinence, which ultimately takes away the bad urine smell resulting in improvement in the social domain [44, 47].

Poor role performance was reported in this study. This finding was similar to the study done in Taiwan [27]. This might be due to the POP affecting women’s QoL negatively by limiting their roles (cleaning, shopping, washing clothes, cooking, fetching water) [48]. The poor personal relationship score in this study was also similar to the study conducted in Turkey [35]. Women with POP in this study also had a poor prolapse impact score. This is similar to studies done in Turkey [35], and the United Kingdom [49]. This might be because annoyance, frustration, and irritation were common in women with POP [15]. The least affected domains were severity measures and sleep/energy. This is similar to the study done in South Africa [32]. This may be due to stress, anxiety, and nocturia in the advanced stage of POP [15]. However, this affected QoL improved following surgery or tissue repair, most likely the result of improved depression symptoms as well as improved bladder symptoms like nocturia [50, 51].

In addition to reporting the burden of poor quality of life among POP patients, the present study was also aimed at identifying factors associated with poor quality of life. Accordingly, having no formal education, having stage III/IV pelvic organ prolapse, constipation, urge urinary incontinence, and no regular physical exercise for at least one/week were independent factors associated with lower pelvic organ-related quality of life.

Women who had no formal education were significantly associated with impaired QoL. These findings go in line with studies done in Bangladesh [17], Bahir Dar [34], and Nepal [45]. This may be because uneducated women incorrectly believed that POP was a tumor, lethal, aging, and incorrect cultural belief was a contextual value for increasing their poor quality of life [34]. Additionally, women who did not get formal education believed that having a hysterectomy would make them feel less feminine and incomplete because the uterus is essential for sexual activity and personality [52]. Women having advanced stage III/IV of POP were significantly associated with poor QoL. This study was supported by a study done in Nepal [13], Bahir Dar [34], Ghana [53], and Thailand [54]. POP symptom increases with advanced stage III/IV, and quality of life reduces [35]. This could be an increased advanced stage III or IV as well as an increased symptom and intensity that adversely impacts women’s social lives, emotional well-being, and functional activities (work, waking, sitting, and standing) [38].

According to this study, constipation was associated with P-QoL. This finding was in line with that of the study conducted in France [55], and the United Kingdom [36]. This might be the severity of the posterior vaginal wall POP-related chronic constipation that prevents emptying capability, which makes them feel emotional, stressed, depressed, and exhausted. [36]. In addition, women with pelvic organ prolapse having constipation experience functional, psychological, and social morbidities, including anxiety, depression, increased somatization, and decreased sexual satisfaction, which could contribute to poor QoL [56]. However, for women diagnosed with POP, posterior and anterior vaginal native tissue regeneration improved the symptoms and avoided negatively impacting the quality of life [57].

Urge urinary incontinence was significantly associated with impaired QoL. These findings were supported by studies done in India [58], and France [55]. This might be a fact that the majority of POP patients experience urge incontinence and suffer from various life impairments, including those related to their psychological health, emotional well-being, social contacts, physical activities, and sexual and interpersonal relationships. [17, 59, 60]. In relation, detrusor overactivity (instability) in women who experience urge incontinence makes them more symptomatic, and insomniac disrupts family relationships and limits their participation in daily activities [15, 61]. However, bladder retraining and avoiding bladder stimulants can alleviate overactive bladder and urge urine incontinence and improve women’s quality of life [62].

Women doing regular physical exercise reduced prolapse impact QoL (meaning that women who didn’t do physical exercise had poor QoL). The finding was supported by the studies done in America [48] and France [55]. This might be because physical exercise strengthens the pelvic floor muscle and improves POP-related symptoms, including stress urinary incontinence, frequency, dribble, and interference with emptying marks general health, physical limitations, emotion, and severity measures were improved [31]. Similarly, physical exercise could increase overall strength, regularly engage pelvic floor musculature, and decrease weight, resulting in good QoL [31, 55, 63].

## Conclusion

More than half of women with POP had a poor quality of life. Poor prolapse quality of life was higher among women who had no formal education, had stage III/IV pelvic organ prolapse, had constipation, had urge urinary incontinence, and did regular physical exercise at least once/week. In addition, the affected women should be offered psychosocial support, early care, and counseling because they have physical, personal, emotional, social, and sleep/energy problems.

Therefore, Gurage Zone Health Bureau with its stakeholders should focus on advocating access to education and promotion for all women, giving evidence-based counseling to do regular physical and pelvic exercises, understanding the significance of illness, identifying its stage, and giving proper care to improve quality of life and early detection of prolapse-related comorbidities like UUI, constipation and give appropriate management to women who have POP to optimize their quality of life.

### Electronic supplementary material

Below is the link to the electronic supplementary material.

Supplementary Material 1

## Data Availability

The datasets analyzed during the current study are available from the corresponding author upon reasonable request.

## Abbreviations

POP: Pelvic Organ Prolapse
QoL: Quality of Life
PRQoL: Prolapse Related Quality of SPSS—Statistical Package for the Social Sciences
