# Supplementary material for: Quality of life and its associated factors among women with pelvic organ prolapse who attend gynecology clinics Southern Ethiopia 2022
Source: BMC Womens Health. 2024 Jul 12;24:398. doi: 10.1186/s12905-024-03238-1 (PMC11241974; doi:10.1186/s12905-024-03238-1)
Supplement: Supplementary file 1 — Supplementary Material 1 [file 12905_2024_3238_MOESM1_ESM.docx]

# Data collection tool

**Annex- I Structured English version questionnaire**

| **Part-1: Sociodemographic characteristics** | | | |
| --- | --- | --- | --- |
| S.No | Variables | Response | Remark |
|  | Age | --------complete years |  |
|  | Residence | 1. Urban 2. Rural |  |
|  | Marital s | 1. Married 2. Single 3. Divorced 4. Widowed |  |
|  | Educational status | 1. Not formally educated 2. Primary education 3. Secondary education and above |  |
|  | Ethnicity | 1. Gurage 2. Amhara 3. Oromo 4. Siltie 5. Others (specify)___________ |  |
|  | Religion | 1. Orthodox 2. Protestant 3. Muslim 4. Catholic 5. Others, specify_________ |  |
|  | Occupation | 1. Housewife 2. Farmer 3. Government employee 4. Private employee 5. Merchant 6. Others (specify) ____ |  |
|  | If married, your husband’s occupation | 1. Farmer 2. Government employee 3. Private employee 4. Merchant 5. Others (specify)________ |  |
|  | Average family monthly income | In ETB--------------------- |  |
| **Part-II Lifestyle and general health condition-related factors of respondent** | | | |
|  | BMI? | ___________in number |  |
|  | Weight | ____________in number |  |
|  | Height | ____________in number |  |
|  | Did you do regular physical exercise (at least 1/week? | 1. Yes 2. No |  |
|  | Did you have self-reported depression? | 1. Yes 2. No |  |
|  | Did you have Hypertension? | 1. Yes 2. No |  |
| **Part-III Obstetrics and gynecology history-related factors of the respondents** | | | |
|  | Gravidity? | _______in number |  |
|  | Parity? | ______ in number |  |
|  | Have you experienced menopause? | 1. Yes 2. No |  |
|  | Have you had a history of previous hysterectomy? | 1. Yes 2. No |  |
|  | Did you have a history of anal surgery? | 1. Yes 2. No |  |
|  | Did you have a history of urinary incontinence surgery? | 1. Yes 2. No |  |
| **Part-IV Pelvic organ prolapse stage and pelvic floor symptom-related questions** | | | |
|  | Pelvic organ prolapse stage | 1. One 2. Two 3. Three 4. Four | Chart review |
|  | Did you experience urge urinary incontinence? (urinary leakage associated with a strong desire to pass urine) | 1. Yes 2. No |  |
|  | Did you experience stress urinary incontinence? (urinary leakage is associated with laughing, coughing, and sneezing) | 1. Yes 2. No |  |
|  | Do you experience frequent urination? | 1. Yes 2. No |  |
|  | Do you experience difficulty emptying your bladder? | 1. Yes 2. No |  |
|  | Do you experience urinary urgency? | 1. Yes 2. No |  |
|  | Do you experience lower abdominal pain or heaviness? | 1. Yes 2. No |  |
|  | Do you experience a feeling of bulging in the vagina? | 1. Yes 2. No |  |
|  | Did you have pain or discomfort with sexual intercourse? | 1. Yes 2. No |  |
|  | Do you ever have difficulty having a bowel movement? | 1. Yes 2. No |  |
|  | Do you experience constipation? (difficulty of defecation) | 1. Yes 2. No |  |
|  | Did you have fecal incontinence? | 1. Yes 2. No |  |

| Part –V Prolapse quality of the life-related question  (A prolapse is a bulge coming down the vagina causing discomfort)  Please fill in this questionnaire even if you feel you don’t have a prolapse. Please tick one answer. | | |
| --- | --- | --- |
|  | **Towards general health perception**  How would you describe your health at present? | Very good Good Fair Poor Very poor |
|  | **Towards pelvic organ prolapse effect**  How much do you think your prolapse problem affects your life? | Not at all Slightly Moderately A lot |
|  | **Towards role limitation**  To what extent does your prolapse affect your household task? (e.g. cleaning, grinding, cooking, washing close, carrying a heavy object)  Does your prolapse affect your daily activity outside the home? (E.g. fetching of water, collection of firewood, selling of grain in the market? | Not at all Slightly Moderately A lot |
|  | **Towards physical limitation**  Does your prolapse affect your physical activity? (e.g. walking, sleeping, standing, sitting)  Does your prolapse affect your ability to travel? | Not at all Slightly Moderately A lot |
|  | **Towards social Limitation**  Does your prolapse limit your social life? (e.g. going to mosque/church)  Does your prolapse limit your ability to see/visit your friend and family? | Not at all Slightly Moderately A lot |
|  | **Towards personal relationship**  Does your prolapse affect your relationship with your partner?  Does your prolapse affect your sex life?  Does your prolapse affect your family life? (e.g. your child, son-in-law? | Not at all Slightly Moderately A lot |
|  | **Towards emotion**  Does your prolapse make you feel depressed?  Does your prolapse make you feel anxious or nervous?  Does your prolapse make you feel bad about yourself? | Not at all Slightly Moderately A lot |
|  | **Towards sleep/energy**  Does your prolapse affect your sleep? (e.g. dream or delirium)  Do you feel worn out/tired? | Never Sometimes Often All the time |
|  | **Towards severity of prolapse**  Do you use panty liners, pads, tampons, and firm knickers to protect prolapse?  Do you push up the prolapse?  Do you have pain or discomfort due to prolapse?  Does the prolapse prevent you from standing? | Never Sometimes Often All the time |
